# Transcriptome profiling reveals characteristics of hairy root and the role of *AhGLK1* in response to drought stress and post-drought recovery in peanut

**DOI:** 10.1186/s12864-023-09219-2

**Published:** 2023-03-16

**Authors:** Xing Liu, Liangchen Su, Limei Li, Zhi Zhang, Xiaoyun Li, Qingjian Liang, Ling Li

**Affiliations:** 1 Department of Bioengineering, Zhuhai Campus of Zunyi Medical University, 519040 Zhuhai, China; 2 Guangdong Provincial Key Laboratory of Biotechnology for Plant Development, School of Life Science, South China Normal University, 510631 Guangzhou, China; 3 School of Fishery, Zhejiang Ocean University, 316022 Zhoushan, Zhejiang China

**Keywords:** Hairy root, AhGLK1, Transcriptome, Drought stress, Post-drought recovery

## Abstract

**Background:**

HR (hairy root) has emerged as a valuable tissue for the rapid characterization of plant gene function and enzyme activity in vivo. AhGLK1 (*Arachis hypogaea* L. golden2-like 1) is known to play a role in post-drought recovery. However, it is unclear (a) whether HR has properties that are distinct from those of PR (primary root); and (b) which gene networks are regulated by AhGLK1 in response to drought stress and recovery in peanut.

**Results:**

We found that cells of the root tip cortex were larger in HR than in PR, while a total of 850 differentially expressed genes (DEGs) were identified in HR compared to PR. Eighty-eight of these DEGs, relating to chlorophyll and photosynthesis, were upregulated in HR. In addition, *AhGLK1-OX* (*AhGLK1*-overexpressing) HR showed a green phenotype, and had a higher relative water content than *35 S::eGFP* (control) HR during drought stress. RNA-seq analysis showed that 74 DEGs involved both in the drought response and the post-drought recovery process were significantly enriched in the galactose metabolism pathway. GO terms enrichment analysis revealed that 59.19%, 29.79% and 17.02% of the DEGs mapped to the ‘biological process’ (BP), ‘molecular function’ (MF) and ‘cellular component’ (CC) domains, respectively. Furthermore, 20 DEGs involved in post-drought recovery were uniquely expressed in *AhGLK1-OX* HR and were significantly enriched in the porphyrin metabolism pathway. GO analysis showed that 42.42%, 30.30% and 27.28% of DEGs could be assigned to the BP, MF and CC domains, respectively. Transcription factors including bHLH and MYB family members may play a key role during drought stress and recovery.

**Conclusion:**

Our data reveal that HR has some of the characteristics of leaves, indicating that HR is suitable for studying genes that are mainly expressed in leaves. The RNA-seq results are consistent with previous studies that show chlorophyll synthesis and photosynthesis to be critical for the role of AhGLK1 in improving post-drought recovery growth in peanut. These findings provide in-depth insights that will be of great utility for the exploration of candidate gene functions in relation to drought tolerance and/or post-drought recovery ability in peanut.

**Supplementary Information:**

The online version contains supplementary material available at 10.1186/s12864-023-09219-2.

## Background

Peanut (*Arachis hypogaea* L.) is an important economic and oil crop that is widely cultivated in arid and semi-arid regions. Its kernels contain about 80% fat and protein, are rich in resveratrol, vitamins, etc., and have certain health care functions. In Asia and Africa, peanut is an important crop for preventing malnutrition and ensuring a safe food supply [1]. Peanut yield and quality are frequently limited because of seasonal drought or fungal disease. Despite the recent availability of a whole genome sequence and increasing amounts of transcriptome data for wild and cultivated peanut varieties, transgenic systems are still not routinely used in peanut plants due to the complexity of the genetic background (tetraploid) and poor efficiency [2]. Chimeras have been generated using tissue culture-independent transformation [3], but selection of lines homozygous for the transgene is time-consuming. Consequently, an alternative, rapid transformation system to facilitate peanut gene characterization is essential and the hairy root (HR) transformation system fulfills this requirement. In previous work, a method for efficient induction of HR from peanut-leaf explants was developed [4, 5], and this has been used to illuminate the molecular function of several genes in peanut, such as *AhHDA1* [6] and *AhGLK1* [7]. HR cultures are particularly easy to grow in hormone-free medium and represent a valuable tool for genetic transformation [8–10], production of high levels of secondary metabolites [11–13], or the regeneration of whole plants [14]. For example, HR has been used to study nodule formation and nitrogen fixation, drought tolerance, nematode interaction, subterranean insects and resveratrol biosynthesis [4, 12, 15, 16]. However, similarities and differences between HR and primary root (PR) have not yet been determined.

Drought is a severe environmental stress that negatively affects vegetative growth. Plants have evolved multiple protective mechanisms that are thought to cooperate to ameliorate adverse environmental factors. Whether crops can produce high yields under drought conditions depends not only on their own drought resistance, but also on their ability to grow during the post-drought recovery phase and to achieve yield compensation after experiencing water deficit. Post-drought recovery and growth have a profound impact on crop yield and quality [17] and represent very complex biological processes, whose mechanisms are completely different from those involved in resistance to drought stress. During the recovery period, in addition to readjusting metabolism to pre-stress levels, plants also need to counteract drought-induced senescence of cells and tissues, which is not simply a return to the pre-stress state. Furthermore, chlorophyll content, photosynthetic rate, antioxidant systems and osmoregulatory compounds are all known to change during drought stress and rehydration [18]. Indeed, many studies have shown that photosynthesis, chlorophyll metabolism, signal transduction, energy metabolism, cell wall synthesis and secondary metabolism are significantly altered during the recovery period following exposure to drought [19–21]. Post-drought recovery is not only highly dependent on the degree of drought stress that plants are subjected to, but also depends on their own innate ability to recover. Studies have shown that peanuts rehydrated after drought before flowering are more productive than those with normal irrigation, and the degree of recovery shows a significant correlation with yield. This may be closely related to the larger root-to-shoot ratio of peanuts during drought. Generally, peanut varieties with strong drought resistance are better able to recover after rehydration. It can be seen that the ability to recover and grow post-drought plays an important role in the yield and quality of peanut, but the molecular mechanisms of recovery are poorly understood.

Golden2-like (GLK) transcription factors are members of the GARP superfamily, and were first reported in maize (*Zea mays* L.) [22]. A large number of studies have confirmed that GLKs regulate the expression of chlorophyll synthesis and photosynthesis-related genes by directly binding to their promoters, thereby affecting chlorophyll synthesis, photosynthesis and chloroplast development [23]. GLKs improve the effective utilization of carbon, increase the accumulation of organic nitrogen, and promote plant growth and development. Furthermore, GLKs are involved in the responses to biotic stressors such as cucumber mosaic virus and turnip yellow mosaic virus [24, 25], as well as some abiotic stresses including ozone and drought [26, 27]. Recent studies have shown that GLKs play an important role in the yield and quality of crops. For example, *SlGLK2* can significantly improve the nutritional content and flavor of tomato [28]. Constitutive expression of maize *GLK* genes in rice results in an increase in the number of seeds per panicle and in grain yield by 20–122% and 30–40%, respectively [29]. In our previous study, when *AhGLK1* was transformed into the *Arabidopsis glk1glk2* mutant, it increased the survival rate of the mutant in post-drought recovery and fully rescued the mutant’s pale-green phenotype [30]. Further studies confirmed that AhGLK1 protein can bind near the transcription start site of the *AhPORA* (*Arachis hypogaea* L. protochlorophyll ideoxidoreductases A) and *AhCAB* (*Arachis hypogaea* L. chlorophyll A/B binding protein) genes to enhance their expression during the post-drought period, thereby promoting recovery growth in peanut after rehydration [7]. However, which gene networks are regulated by AhGLK1 in response to drought stress and recovery in peanut is unknown.

This study aimed to provide more details on the differences between PR and HR, and to generate more extensive data than previous analyses on the drought stress and post-drought recovery responses of peanut HR. Moreover, we wanted to identify unique candidate genes involved in *AhGLK1*-overexpressing HR (*AhGLK1-OX* HR) that might enhance its drought resistance and ability to recover. To this purpose, we first identified similarities and differences between peanut HR and PR in both root morphology and RNA-seq profile. Secondly, we performed RNA-seq analysis under control, drought and recovery conditions for 30-day-old *35 S::eGFP* (blank control group) and *AhGLK1-OX* HR to screen for *AhGLK1*-controlled gene networks involved in the response to drought and in post-drought recovery. The results show that there is marked genetic plasticity in the response to drought stress and recovery in *35 S::eGFP* and *AhGLK1-OX* HR, which provides valuable information for the clarification of regulatory mechanisms of drought-response and post-drought recovery processes in peanut.

## Results

### Structural characteristics of hairy root and primary root

**Fig. 1:**
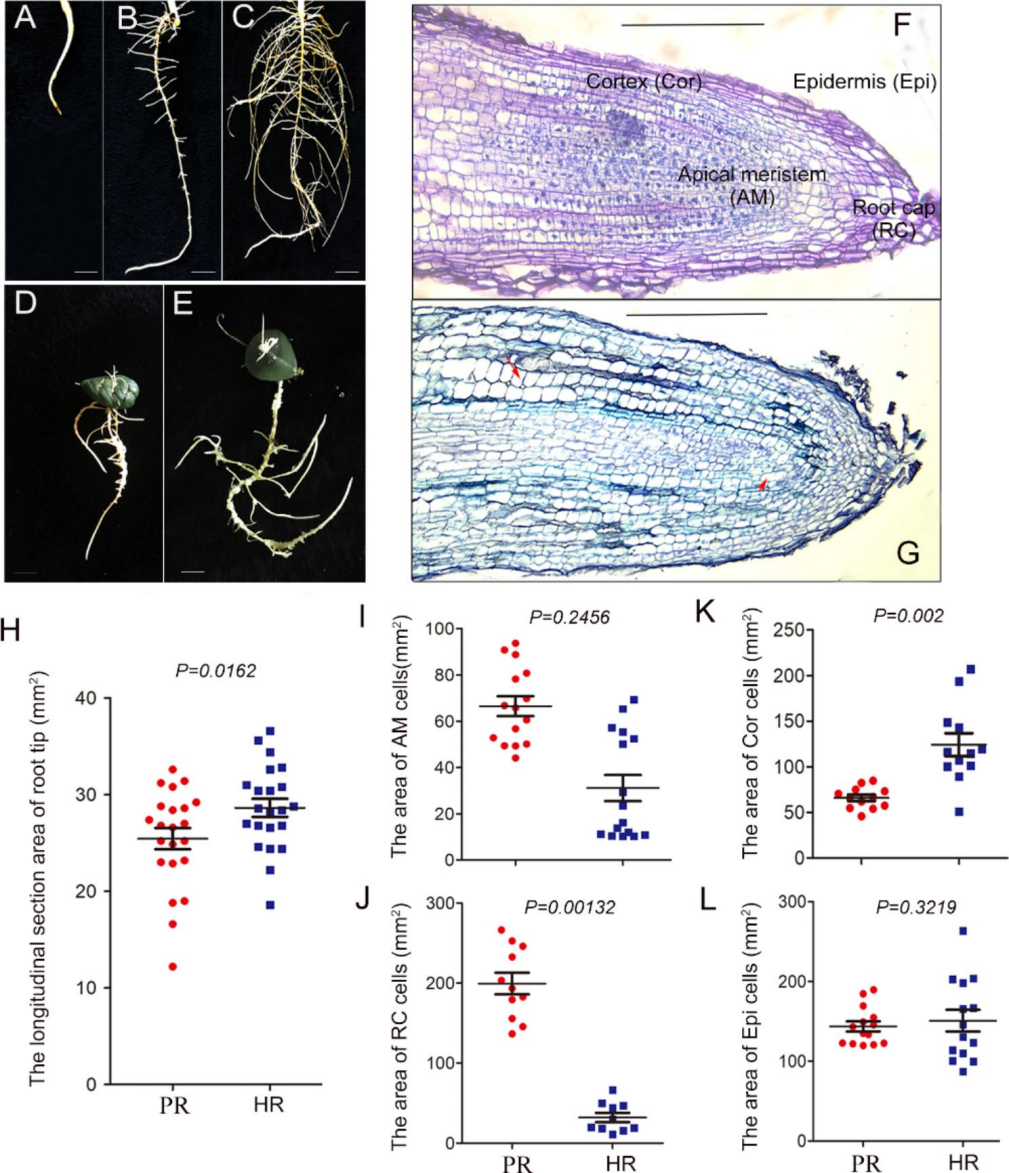
**Structural characteristics of HR and PR.** (A-C) show the PR of peanut from germination to two-leaf stage. (D-E) show HR at early (< 20 days) and late (> 30 days) stages. (F-G) indicate the structure of the tip of PR (F) and HR (G) viewed through an upright fluorescence microscope. (H) Comparison of the longitudinal section area of PR and HR tips. (I-L) indicate the area of apical meristem, root tip, cortex and epidermis cells of PR and HR. Both t-tests and F-tests were conducted using PRISM5 to compare variance between PR and HR. The *P*-value (F-test) is shown in the diagram, where *P* < 0.05 is taken to indicate a significant difference

HR was induced as previously described [5] using peanut leaf explants infected with *A. rhizogenes*. To explore the structural characteristics of HR, we compared HR (grown for 20 or 30 days) and PR (grown for 14 days). As seedling growth proceeded, the PR became elongated with extensive lateral roots (Fig. 1A-C). In contrast, HR was induced at most wound sites, and some roots grew as long as PR, although lateral HR was present in different numbers (Fig. 1D-E). Under the light microscope, the surface of the PR tip was very smooth, because the meristem cells regularly undergo division and gradually differentiate into root cap, epidermis or elongation zone. The root cap, epidermis and zone of elongation consisted of large rectangular cells, while the cortex and meristem contained small square cells (Fig. 1F). HR also showed differentiation and maturation into root cap, epidermis or elongation zone (Fig. 1G), but the root tip longitudinal section area was larger than in PR (Fig. 1H). The cells of the root cap and apical meristem were smaller in HR than in PR (Fig. 1I-J); in contrast, the cells of the cortex were larger in HR than in PR (Fig. 1K). No significant difference was observed between the epidermis cells of the two root types (Fig. 1L). Taken together, we speculate that the inconsistency in cell size in the cortex results in the HR being abnormally enlarged.

### Transcriptome analysis of hair root and primary root

**Fig. 2:**
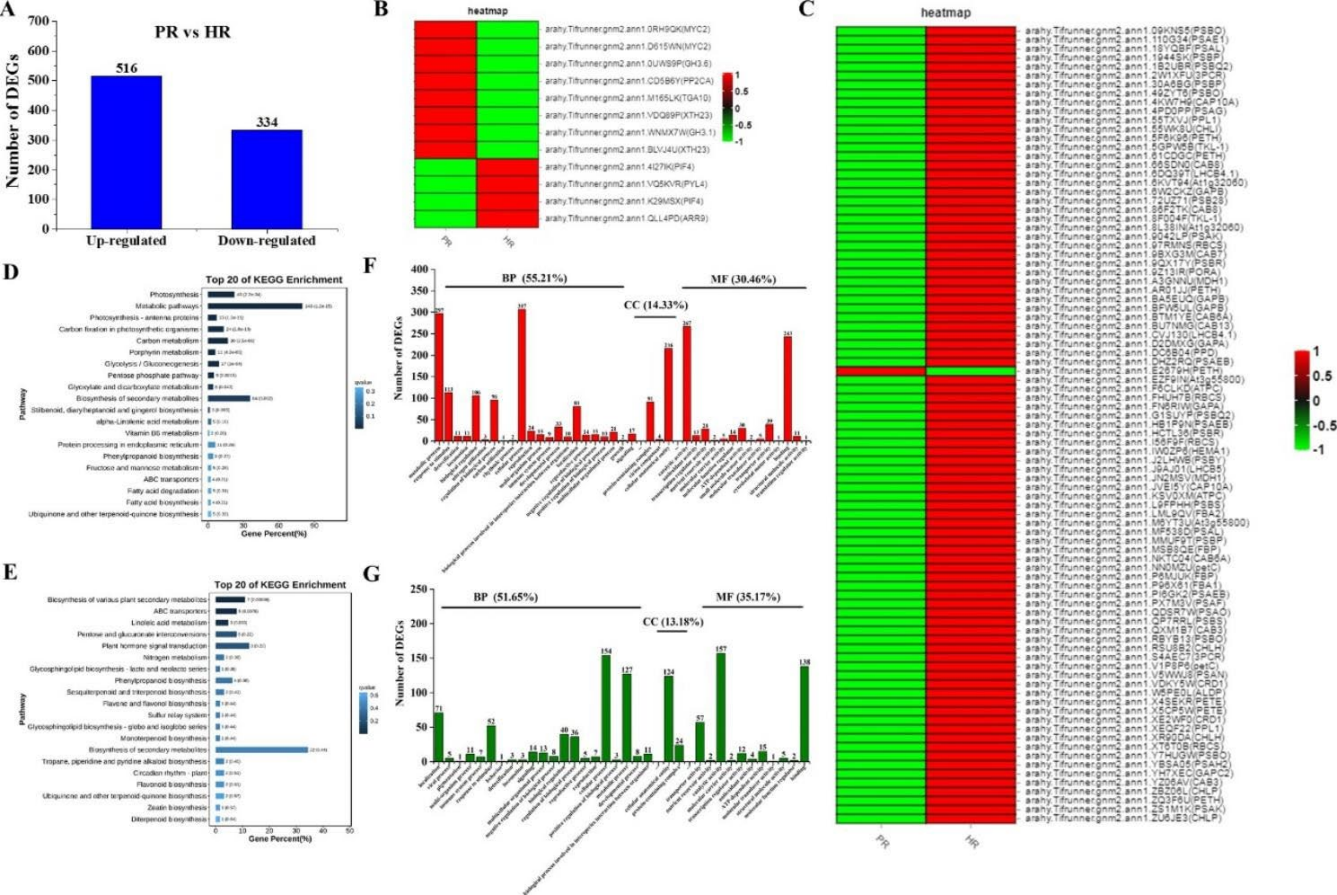
**Analysis of genes that are differentially expressed in HR compared to PR.** (A) The number of upregulated and downregulated DEGs in HR compared to PR. (B) Heatmap analysis showing hormone signal transduction DEGs in HR and PR. (C) Heatmap analysis showing photosynthesis and chlorophyll-related DEGs in HR and PR. (D) Top twenty enriched KEGG pathways to which upregulated DEGs map. (E) Top twenty enriched KEGG pathways to which downregulated DEGs map. (F) GO enrichment analysis of upregulated DEGs. (G) GO enrichment analysis of downregulated DEGs.

HR exhibits many of the functions of PR, such as nodule formation and nitrogen fixation, interaction with nematodes and biosynthesis of resveratrol. However, as shown above, there are clear differences in the structural anatomy of HR and PR. To understand these differences at the genetic level, we compared the global transcriptomes of HR and PR and found that 850 genes were significantly differentially expressed between the two types of root. Of these, 516 genes were upregulated and 334 genes were downregulated in HR compared to PR (Fig. 2A; Supplemental Datasets 1 and 2). Interestingly, 8 DEGs relating to hormone signal transduction were downregulated and 87 DEGs relating to chlorophyll and photosynthesis were upregulated in HR compared to PR. Heatmap analysis further confirmed these results (Fig. 2B and C). Together, these data suggest that hormone signal transduction pathways are different in HR, and that HR possesses at least some of the characteristics of leaves, such as the synthesis of chlorophyll and photosynthesis. Therefore, we considered that HR might be suitable for the study of genes that are mainly expressed in leaves.

We investigated the DEGs in more detail by subjecting the upregulated DEGs to KEGG pathway enrichment analysis and GO term enrichment analysis. As shown in Fig. 2D, the top 20 KEGG pathways included metabolic pathways (143 DEGs), biosynthesis of secondary metabolites (64 DEGs), photosynthesis and photosynthesis-antenna proteins (53 DEGs), carbon fixation in photosynthetic organisms (24 DEGs), carbon metabolism (30 DEGs) and porphyrin metabolism (11 DEGs). Photosynthesis and chlorophyll-related pathways accounted for 17.12% of the total, which was consistent with the fact that many DEGs had functions that are likely associated with chlorophyll synthesis and photosynthesis, further confirming that HR shows similar functionality to leaves.

Downregulated DEGs were significantly enriched in KEGG pathways involving biosynthesis of various plant secondary metabolites (7 DEGs), ABC transporters (5 DEGs) and linoleic acid metabolism (3 DEGs). The plant hormone signal transduction pathway was also enriched, with 8 DEGs assigned to it (Fig. 2E).

GO term enrichment analysis showed that 55.21% of the upregulated DEGs and 51.65% of the downregulated DEGs mapped to the domain ‘biological process’. In the case of upregulated DEGs, those mapping to ‘biological process’ predominantly included the terms ‘cellular process’, ‘metabolic process’ and ‘response to stimulus’. Downregulated DEGs mapping to ‘biological process’ showed a slightly different profile, being predominantly involved in ‘cellular process’, ‘metabolic process’ and ‘localization’. In the ‘cellular component’ category, 14.33% and 13.18% of up- and downregulated DEGs, respectively, corresponded to the terms ‘cellular anatomical entity’ and ‘protein-containing complex’. Finally, in the ‘molecular function’ category, 30.46% and 35.17% of up- and downregulated DEGs, respectively, corresponded to the terms ‘catalytic activity’, ‘binding’ and ‘transporter activity’ (Fig. 2F and G).

### Phenotype of *AhGLK1-OX* hairy root

**Fig. 3:**
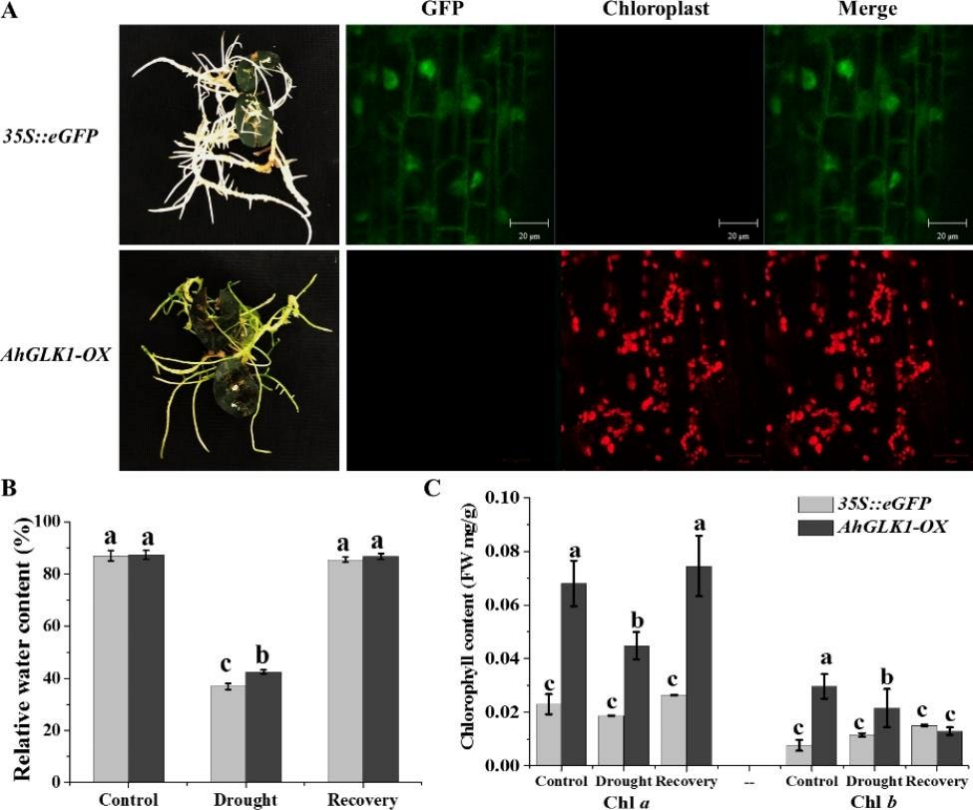
**Phenotypic characteristics of*35S::eGFP*and*AhGLK1-OX*hairy root** (A) Phenotype of *35 S::eGFP* and *AhGLK1-OX* HR by naked eye and confocal microscopy. (B) The relative water content of the two types of HR under different conditions. (C) Chlorophyll contents of the two types of HR under different conditions. The average of three biological replicates is shown. Error bars represent SE. Different letters (a, b, c) represent significantly different groups

Previously, we demonstrated that the HR transformation system represents an invaluable research tool in peanut for the investigation of gene function during drought stress [5]. In addition, *AhGLK1* was confirmed to play an important role in post-drought recovery by regulating the expression of chlorophyll synthesis and photosynthesis-related genes [7, 30]. Here, to understand in more detail the function of *AhGLK1* during drought and post-drought recovery in peanut, we first analyzed the phenotype of *AhGLK1-OX* HR. As shown in Fig. 3A, *AhGLK1-OX* HR appeared green, and many chloroplasts were observed by confocal microscopy. Interestingly, while after dehydration for 2 h the relative water content of both *35 S::eGFP* and *AhGLK1-OX* HR was significantly reduced compared to the control, the relative water content of *AhGLK1-OX* HR was higher than that of *35 S::eGFP* HR, indicating that *AhGLK1-OX* HR has better drought tolerance. After rehydration for 2 h, there was no significant difference in the relative water content of the two types of HR (Fig. 3B). Furthermore, the Ch *a* content of *AhGLK1-OX* HR was clearly higher than that of *35 S::eGFP* HR after both dehydration and rehydration for 2 h, as well as in control plants. However, although when dehydrated for 2 h Ch *b* content of *AhGLK1-OX* HR was higher than that of *35 S::eGFP*, there was no significant difference after rehydration for 2 h (Fig. 3C). The results indicate that Ch *a* and Ch *b* of *AhGLK1-OX* HR varied differently during drought stress and recovery.

### Transcriptome analysis of the hair root response to drought stress

**Fig. 4:**
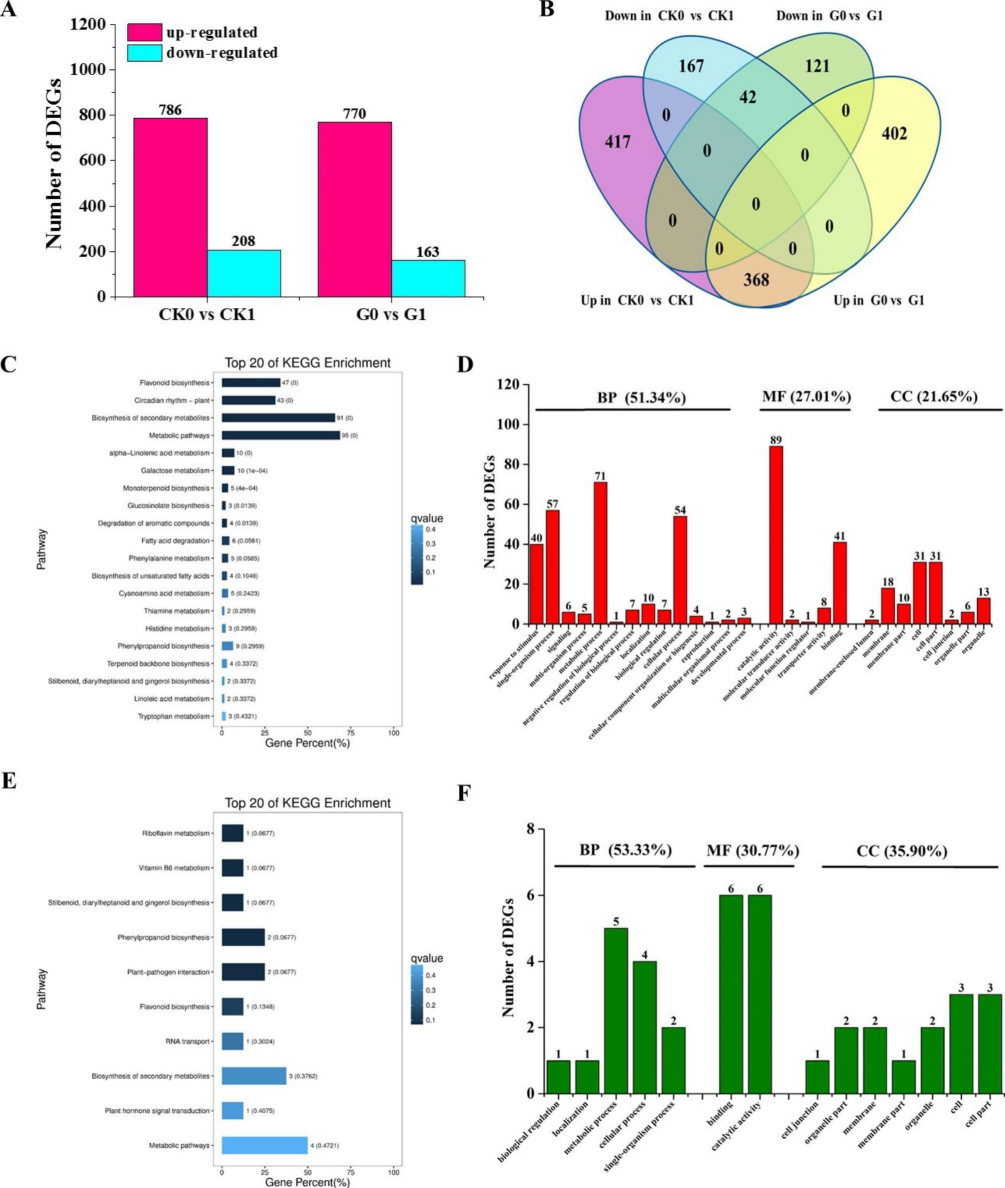
**Analysis of genes that are differentially expressed in*35S::eGFP*and*AhGLK1-OX*hairy root during drought stress.** (A) The number of DEGs in the two pairwise groups. (B) Venn diagram of common and uniquely expressed genes for the pairwise comparisons CK0 vs. CK1 and G0 vs. G1. (C) Top twenty enriched KEGG pathways to which DEGs upregulated during drought stress map. (D) GO enrichment analysis of upregulated DEGs assigned to the domains ‘biological process’ (BP), ‘cellular component’ (CC) and ‘molecular function’ (MF). (E) Top twenty enriched KEGG pathways to which DEGs downregulated during drought stress map. (F) GO enrichment analysis of downregulated DEGs assigned to the domains ‘biological process’ (BP), ‘cellular component’ (CC) and ‘molecular function’ (MF).

To obtain detailed information about the genes involved in the drought stress response, we compared the expression profiles of *35 S::eGFP* and *AhGLK1-OX* HR during control conditions, drought stress and (next section) post-drought recovery. In the following sections, samples are denoted CK0 (control conditions, *35 S::eGFP* HR), CK1 (drought, *35 S::eGFP* HR), CK2 (recovery, *35 S::eGFP* HR), G0 (control, *AhGLK1-OX* HR), G1 (drought, *AhGLK1-OX* HR) and G2 (recovery, *AhGLK1-OX* HR). Sequence reads that passed quality filtering were used for reference-based alignment using the TopHat pipeline. A total of 89.76–93.55% of the total Illumina reads aligned to the reference genome. Uniquely aligned reads were used to estimate gene expression levels as FPKM. For further analysis of expression patterns, only statistically significant DEGs from each sample were used. For the comparison of CK0 with CK1, we identified 786 and 208 genes that were up- and downregulated, respectively, while 770 and 163 genes were up- and downregulated when G0 and G1 were compared (Fig. 4A; Supplemental Datasets 3 and 4). These DEGs were depicted in a Venn diagram (Fig. 4B). There were 368 upregulated DEGs in common between CK0 vs. CK1 and G0 vs. G1, indicating that these genes are involved in the response to drought stress both in *35 S::eGFP* and *AhGLK1-OX* HR. In contrast, 417 and 402 uniquely upregulated genes were found for CK0 vs. CK1 and G0 vs. G1, respectively. We also analyzed downregulated genes for the two pairwise comparisons and found 42 downregulated DEGs in common between CK0 vs. CK1 and G0 vs. G1. In contrast, 167 and 121 uniquely downregulated genes were found for CK0 vs. CK1 and G0 vs. G1, respectively (Fig. 4B; Supplemental Datasets 5 and 6). It is striking that there are many more DEGs in common in the upregulated category (i.e. 368 vs. 42), implying that these genes are induced by drought stress.

To further understand the biological pathways that are activated or inhibited by drought stress, KEGG pathway enrichment analysis was performed for each pairwise group to assess the extent of regulation of the DEGs. Nine out of the top 20 KEGG pathways that were significantly enriched in upregulated DEGs comprised flavonoid biosynthesis (47 DEGs), circadian rhythm–plant (43 DEGs), biosynthesis of secondary metabolites (91 DEGs) and metabolic pathways (95 DEGs) (Fig. 4C). The results suggest that these pathways play a key role in the response to drought stress. GO analysis was used for the functional classification of these DEGs and showed that 51.34% mapped to ‘biological process’, predominantly involving the terms ‘metabolic process’, ‘single-organism process’, ‘cellular process’ and ‘response to stimulus’. Approximately 27% of the DEGs mapped to the ‘molecular function’ domain, mainly corresponding to ‘catalytic activity’ and ‘binding’, while 21.65% corresponded to the ‘cellular component’ domain, including ‘cell’, ‘cell part’ and ‘membrane’ (Fig. 4D). Among downregulated DEGs, only 10 KEGG pathways were enriched, but none significantly (Fig. 4E). As shown in Fig. 4F, similarly to the upregulated DEGs, 53.33% of the downregulated DEGs could be assigned to the ‘biological process’ domain, predominantly in the ‘metabolic process’ and ‘cellular process’ categories. Approximately 31% of the downregulated DEGs mapped to the ‘molecular function’ domain, mainly corresponding to the ‘catalytic activity’ and ‘binding’ categories, while 35.90% of the downregulated DEGs mapped to the ‘cellular component’ domain, including ‘membrane’, ‘cell’ and ‘cell part’ among the predominant terms.

### Transcriptome analysis of hair root in post-drought recovery

**Fig. 5:**
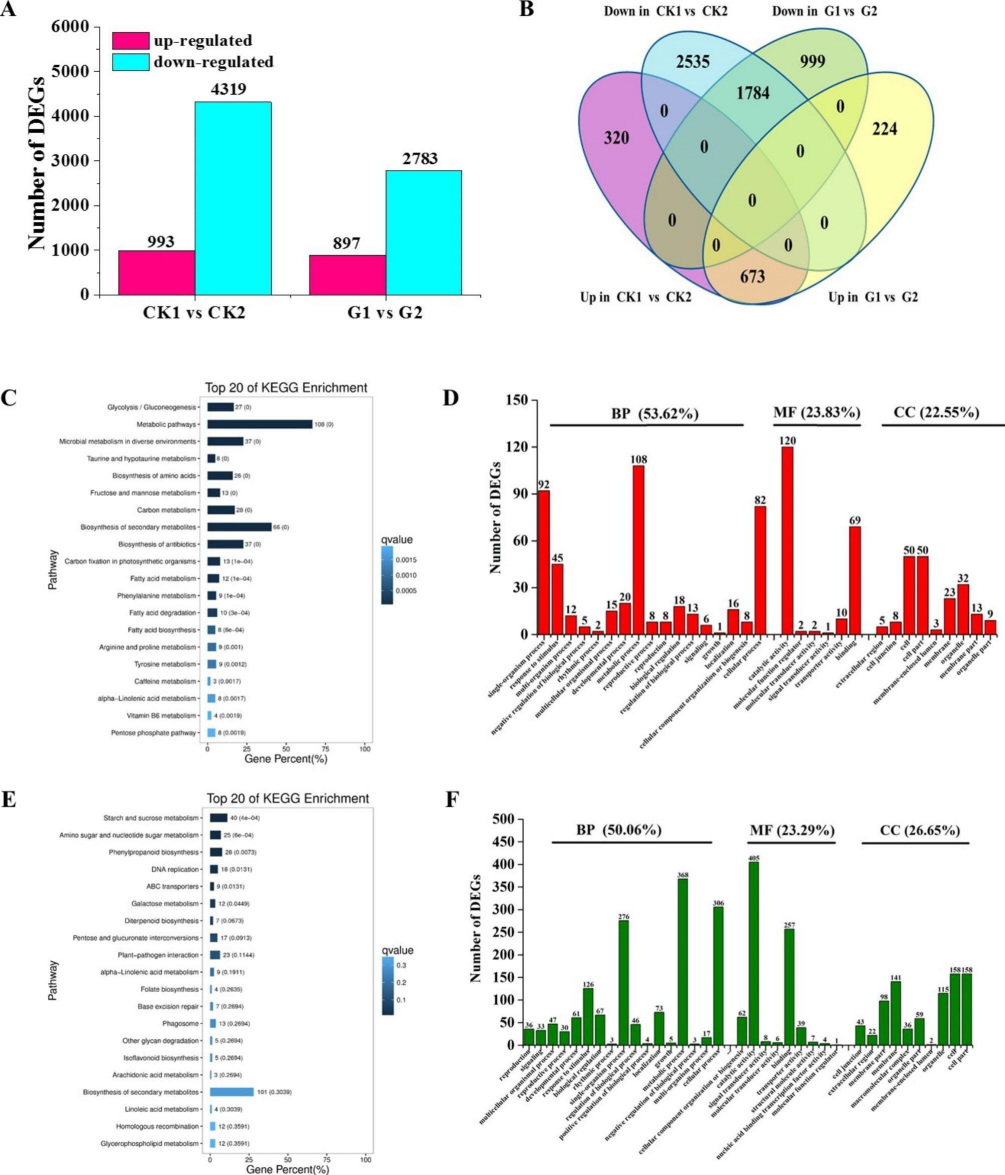
**DEG analysis of*35S::eGFP*and*AhGLK1-OX*hairy root in post-drought recovery.** (A) The number of DEGs in the two pairwise groups. (B) Venn diagram of common and uniquely expressed genes for the pairwise comparisons CK1 vs. CK2 and G1 vs. G2. (C) Top twenty enriched KEGG pathways to which DEGs upregulated in post-drought recovery map. (D) GO enrichment analysis of upregulated DEGs assigned to the domains ‘biological process’ (BP), ‘cellular component’ (CC) and ‘molecular function’ (MF). (E) Top twenty enriched KEGG pathways to which DEGs downregulated in post-drought recovery map. (F) GO enrichment analysis of downregulated DEGs assigned to the domains ‘biological process’ (BP), ‘cellular component’ (CC) and ‘molecular function’ (MF).

We next compared the expression profiles of *35 S::eGFP* and *AhGLK1-OX* HR in post-drought recovery. For CK1 vs. CK2, we identified 993 and 4319 genes that were up- and downregulated, respectively, while 897 and 2783 genes were up- and downregulated, respectively, when G1 and G2 were compared (Fig. 5A; Supplemental Datasets 7 and 8). As shown in the Venn diagram (Fig. 5B), there were 673 upregulated DEGs in common between CK1 vs. CK2 and G1 vs. G2, indicating that these genes are involved in the response to post-drought recovery both in *35 S::eGFP* and *AhGLK1-OX* HR. In contrast, 320 and 224 uniquely upregulated genes were found for CK1 vs. CK2 and G1 vs. G2, respectively. We also analyzed downregulated genes for the two pairwise comparisons and found 1784 downregulated DEGs in common. In contrast, 2535 and 999 uniquely downregulated genes were found in the two pairwise groups, respectively (Fig. 5B; Supplemental Datasets 9 and 10). Thus, most of the DEGs identified were downregulated during the recovery process.

KEGG pathway analysis showed that metabolic pathways (108 DEGs) and biosynthesis of secondary metabolites (66 DEGs) were predominant among the top 20 enriched pathways (Fig. 5C), suggesting that these two activities are the most important during post-drought recovery, when genes involved in these pathways may be upregulated. We also carried out GO term enrichment analysis. As shown in Figs. 5D and 53.62% of DEGs mapped to ‘biological process’, predominantly involving the terms ‘metabolic process’, ‘single-organism process’, ‘cellular process’ and ‘response to stimulus’. Approximately 24% of DEGs could be assigned to ‘molecular function’, mainly corresponding to ‘catalytic activity’ and ‘binding’, while 22.55% of DEGs mapped to the ‘cellular component’ domain, including the terms ‘cell’, ‘cell part’ and ‘organelle’. For downregulated DEGs, 16 out of the top 20 KEGG pathways were significantly enriched. Starch and sucrose metabolism (40 DEGs), amino sugar and nucleotide sugar metabolism (25 DEGs) and phenylpropanoid biosynthesis (28 DEGs) were the most significantly enriched (Fig. 5E), indicating that these pathways were inhibited during post-drought recovery. Similarly to the upregulated DEGs, 50.06% of downregulated DEGs could be assigned to the GO ontology ‘biological process’, predominantly including ‘metabolic process’, ‘cellular process’, ‘single-organism process’ and ‘response to stimulus’. Approximately 24% of the downregulated DEGs mapped to ‘molecular function’, mainly comprising ‘catalytic activity’, ‘binding’ and ‘cellar component organization or biogenesis’, while 26.65% of the downregulated DEGs were assigned to the ‘cellular component’ domain, including the terms ‘cell’, ‘cell part’, ‘membrane’ and ‘organelle’ (Fig. 5F).

### Transcriptome analysis reveals AhGLK1-regulated gene networks during drought stress and post-drought recovery

**Fig. 6:**
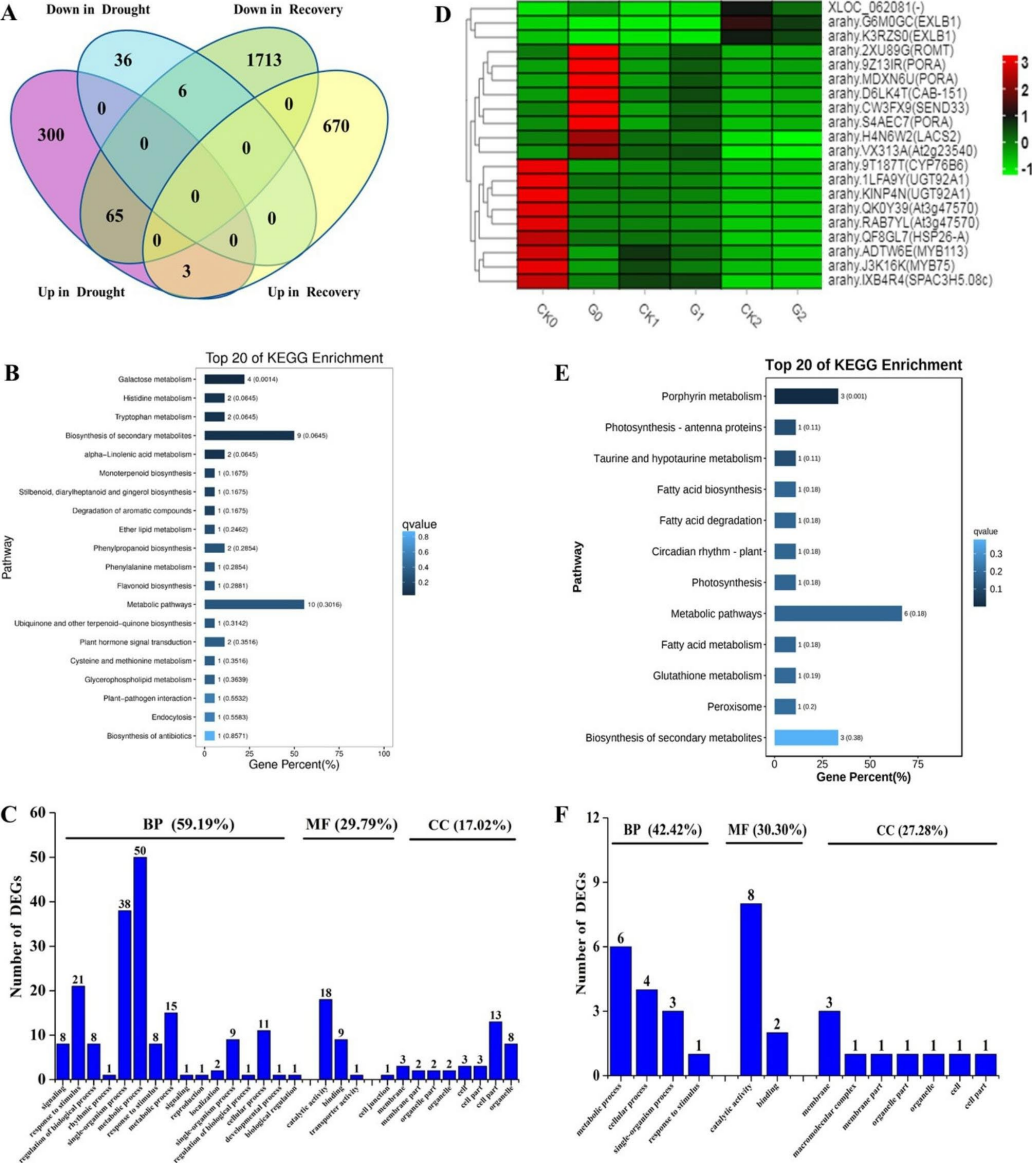
**Analysis of DEGs common to the response to drought stress and post-drought recovery in*35S::eGFP*and*AhGLK1-OX*hairy root.** (A) Venn diagram of common and uniquely expressed genes during drought and post-drought recovery. (B) Top twenty KEGG pathways enriched in common DEGs expressed during drought and post-drought recovery. (C) GO enrichment analysis of common DEGs expressed in drought and post-drought recovery assigned to the domains ‘biological process’ (BP), ‘cellular component’ (CC) and ‘molecular function’ (MF). (D) Heatmap analysis showing expression of the 20 DEGs in the different samples. (E) Top twenty KEGG pathways enriched for DEGs uniquely expressed in *AhGLK1-OX* HR, but commonly expressed during drought and recovery. (F) GO enrichment analysis of DEGs uniquely expressed in *AhGLK1-OX* HR, but commonly expressed during drought and recovery, mapping to the domains ‘biological process’ (BP), ‘cellular component’ (CC) and ‘molecular function’ (MF).

To identify AhGLK1-regulated gene networks that operate during drought stress and post-drought recovery, we first analyzed the DEGs that were common to the response to drought stress and post-drought recovery. As shown in Figs. 6A and 74 DEGs formed part of both the drought-stress response and post-drought recovery processes (Supplemental Dataset 11), suggesting that these DEGs are important in both phases in *35 S::eGFP* and *AhGLK1-OX* HR. Among the 74 DEGs, three and six DEGs were upregulated and downregulated, respectively, during both drought stress and post-drought recovery, indicating that these gene functions may need to increase or decrease under both conditions. We found that 65 DEGs were upregulated in drought but downregulated in recovery, suggesting that their functions are necessary for the drought stress response, but are less important for recovery after drought.

We subjected the 74 DEGs to KEGG pathway and GO term enrichment analysis. KEGG pathway analysis showed that the DEGs were significantly enriched in the galactose metabolism pathway. It is worth noting that the plant hormone signal transduction and flavonoid biosynthesis pathways were also enriched (Fig. 6B). GO analysis revealed that 59.19% of DEGs mapped to ‘biological process’, with major terms being ‘metabolic process’, ‘single-organism process’ and ‘response to stimulus’. Approximately 30% of DEGs could be assigned to the ‘molecular function’ domain, mainly corresponding to ‘catalytic activity’ and ‘binding’, while 17.02% of the DEGs mapped to the ‘cellular component’ domain, including ‘cell part’ and ‘organelle’ among the main terms identified (Fig. 6C).

To identify which genes expressed in *AhGLK1-OX* HR were involved in the response to drought stress and recovery, we also investigated genes that were differentially expressed between CK0 vs. G0, as well as the DEGs that were common to both the drought-stress response and recovery. We found 20 DEGs that were uniquely expressed in *AhGLK1-OX* HR, but commonly expressed in recovery (Supplemental Dataset 12). Of these, three and 17 DEGs were upregulated and downregulated in recovery, respectively. We also noted that eight DEGs that were downregulated in recovery were upregulated in *AhGLK1-OX* HR, whereas nine DEGs downregulated in recovery were also downregulated in *AhGLK1-OX* HR (Fig. 6D). We consider these genes might play an important role in the recovery of *AhGLK1-OX* HR after drought stress.

Next, we performed KEGG pathway and GO term enrichment analysis. KEGG pathway analysis showed that the metabolic, photosynthesis-antenna proteins and photosynthesis, and porphyrin metabolism pathways were enriched (Fig. 6E). The result suggests that photosynthesis and chlorophyll synthesis are important for *AhGLK1-OX* HR in response to stresses in the external environment, consistent with a previous study [7]. GO analysis showed that 42.42% of DEGs were involved in ‘biological process’, including the terms ‘metabolic process’, ‘cellular process’, ‘single-organism process’ and ‘response to stimulus’. Approximately 30% of DEGs mapped to the ‘molecular function’ domain, with prominent terms being ‘catalytic activity’ and ‘binding’, while 27.28% of DEGs could be assigned to the ‘cellular component’ domain, mainly involving the term ‘membrane’ (Fig. 6F).

### Transcriptome analysis of *35 S::eGFP* hair root in response to drought and post-drought recovery

**Fig. 7:**
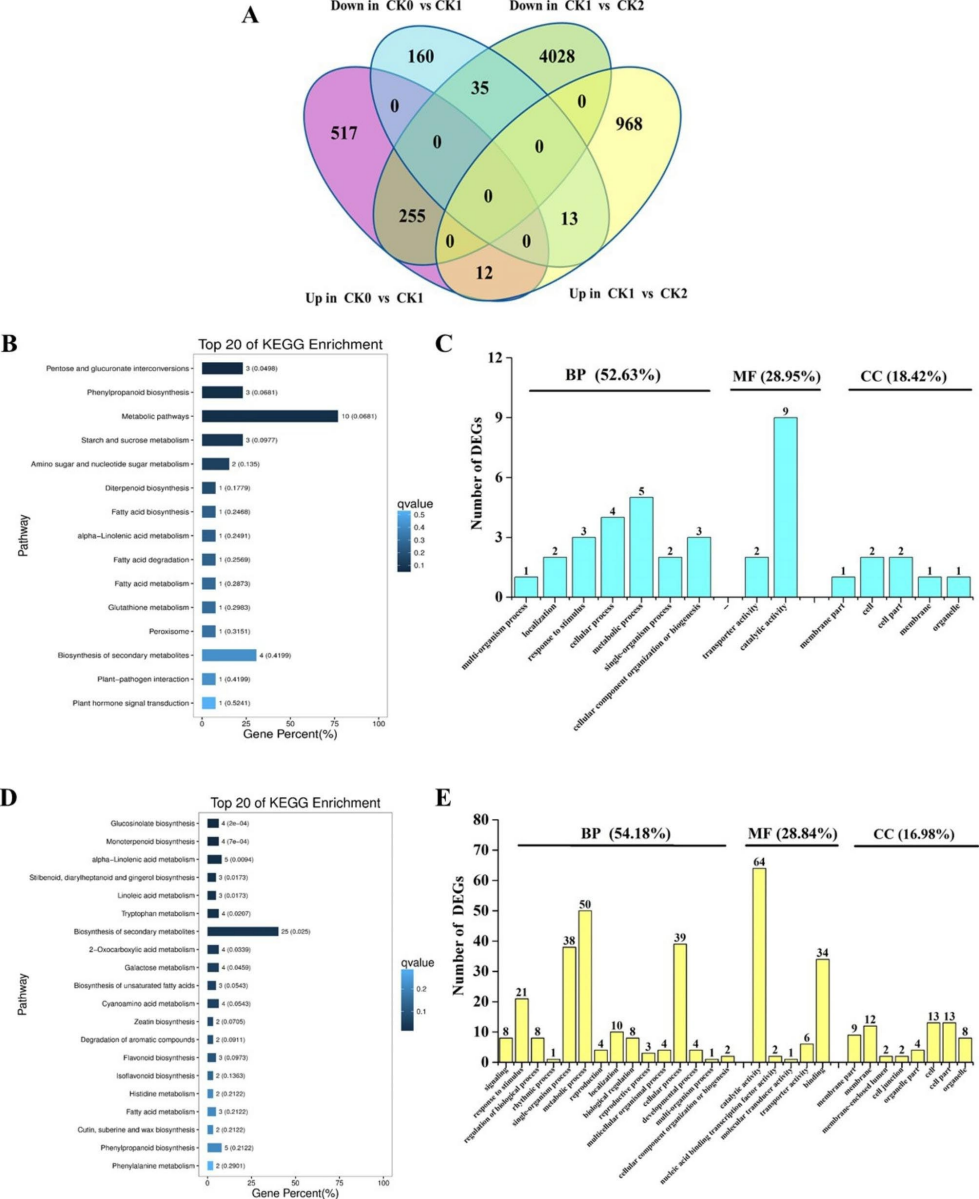
**Analysis of DEGs identified in*35S::eGFP*hairy root during drought and post-drought recovery.** (A) Venn diagram of common and unique DEGs identified in the pairwise CK0 vs. CK1 and CK1 vs. CK2 comparisons. (B) Top twenty KEGG pathways enriched in up- or downregulated DEGs. (C) GO analysis mapping up- or downregulated DEGs to the ‘biological process’ (BP), ‘cellular component’ (CC) and ‘molecular function’ (MF) domains. (D) Top twenty KEGG pathways enriched in DEGs expressed in opposite patterns during drought and recovery. (E) GO analysis of DEGs expressed in opposite patterns during drought and recovery showing assignment to the ‘biological process’ (BP), ‘cellular component’ (CC) and ‘molecular function’ (MF) domains

We also analyzed the transcriptome profile of *35 S::eGFP* HR under drought stress and recovery conditions. By comparing the transcriptome profiles of CK0 vs. CK1 and CK1 vs. CK2, a total of 677 DEGs (517 upregulated and 160 downregulated) were uniquely and uniformly identified by comparison of CK0 with CK1 only, whereas a total of 4996 DEGs (968 upregulated and 4028 downregulated) resulted from comparing CK1 with CK2. Furthermore, 12 and 35 DEGs were commonly found to be upregulated and downregulated for the pairwise comparisons CK0 vs. CK1 and CK1 vs. CK2, respectively. In addition, 255 DEGs showed the opposite gene expression pattern in CK0 vs. CK1 and CK1 vs. CK2, i.e., the genes were upregulated in the CK0 vs. CK1 comparison, but downregulated in CK1 vs. CK2. Similarly, 13 DEGs were downregulated for CK0 vs. CK1, but upregulated for CK1 vs. CK2 (Fig. 7A; Supplemental Dataset 13). The results suggest that 315 DEGs are involved in the response to drought stress and post-drought recovery in *35 S::eGFP* HR. Most of these were induced by drought, but repressed during the recovery process.

These 315 DEGs were then allocated to different functional groups to facilitate further analysis on gene expression. The annotations of the DEGs from both CK0 vs. CK1 and CK1 vs. CK2 pairwise comparisons were checked carefully and integrated using KEGG pathway and GO classification. The 47 common up- or downregulated DEGs were significantly enriched in the pentose and glucoronate interconversions pathway (Fig. 7B). The top GO terms mainly involved ‘biological process’, which was indicated for 52.63% of the DEGs, including the terms ‘metabolic process’, ‘cellular process’ and ‘response to stimulus’. Approximately 29% of the DEGs mapped to the ‘molecular function’ domain, mainly corresponding to the terms ‘catalytic activity’ and ‘transporter activity’, while 18.42% of the DEGs could be assigned to the ‘cellular component’ domain, including the terms ‘cell’ and ‘cell part’ (Fig. 7C).

We also analyzed the DEGs that showed an opposite gene expression pattern during drought and recovery. The 268 DEGs were significantly enriched in 11 out of the top 20 KEGG pathways. Metabolic pathways were the most significantly enriched, including alpha-linolenic acid metabolism, linolenic acid metabolism, tryptophan metabolism, biosynthesis of secondary metabolism, 2-oxocarboxylic acid metabolism and galactose metabolism. In addition, the glucosinolate biosynthesis pathway was also significantly enriched (Fig. 7D). GO analysis showed that 54.18% of DEGs were assigned to the ‘biological process’ domain, with the main terms being ‘metabolic process’, ‘single-organism process’, ‘cellular process’ and ‘response to stimulus’. Approximately 20% of the DEGs mapped to ‘molecular function’, mainly corresponding to ‘catalytic activity’ and ‘binding’, while only 16.98% of the DEGs were assigned to the ‘cellular component’ domain, with the main terms including ‘cell’, ‘cell part’ and ‘membrane’ (Fig. 7E).

### Transcriptome analysis of *AhGLK1-OX* hairy root in response to drought and post-drought recovery

**Fig. 8:**
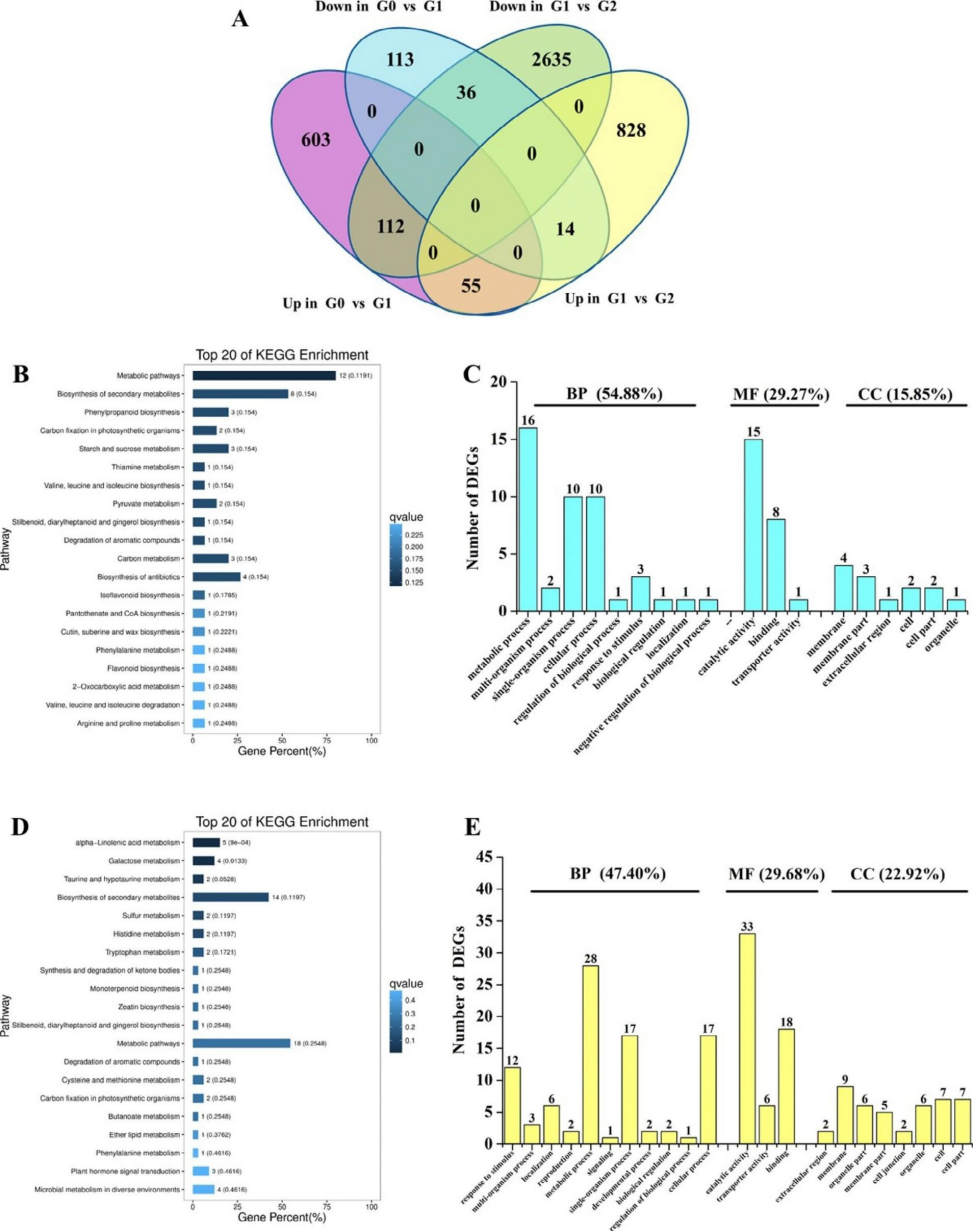
**Analysis of DEGs in*AhGLK1-OX*hairy root in response to drought and post-drought recovery.** (A) Venn diagram of common and uniquely expressed genes identified by G0 vs. G1 and G1 vs. G2 comparisons. (B) Top twenty KEGG pathways enriched in up- or downregulated DEGs. (C) GO analysis mapping up- or downregulated DEGs to the ‘biological process’ (BP), ‘cellular component’ (CC) and ‘molecular function’ (MF) domains. (D) Top twenty KEGG pathways enriched in DEGs expressed in opposite pattern during drought and recovery. (E) GO analysis of DEGs expressed in opposite patterns during drought and recovery showing assignment to the ‘biological process’ (BP), ‘cellular component’ (CC) and ‘molecular function’ (MF) domains

To explore the details of genes involved in the response to drought stress and post-drought recovery in *AhGLK1-OX* HR, we compared the transcriptome profiles of G0 vs. G1 and G1 vs. G2. A total of 716 DEGs (603 upregulated and 113 downregulated) were uniquely and uniformly expressed in G0 vs. G1 only, whereas a total of 3463 DEGs (828 upregulated and 2635 downregulated) were uniquely found in the comparison of G1 with G2. In addition, 55 and 36 upregulated and downregulated DEGs, respectively, were common to both the G0 vs. G1 and G1 vs. G2 comparisons. In contrast, 126 DEGs showed the opposite gene expression pattern in the two pairwise comparisons. Among this last group of DEGs, 112 DEGs were upregulated during drought but downregulated during the recovery process, whereas 14 DEGs were downregulated during drought but upregulated during recovery (Fig. 8A; Supplemental Dataset 14), implying that their expression responds to whether drought or recovery conditions prevail.

To gain insight into the function of the 207 DEGs identified under drought stress and post-drought recovery conditions, KEGG pathways and GO terms were analyzed. First, we found that the up- or downregulated DEGs that were common to both the G0 vs. G1 and G1 vs. G2 comparisons were mainly enriched in metabolic and biosynthesis pathways (Fig. 8B). GO analysis showed that 54.88% of DEGs mapped to the domain ‘biological process’, with predominant terms including ‘metabolic process’, ‘single-organism process’ and ‘cellular process’. Approximately 29% of DEGs could be assigned to the ‘molecular function’ domain, mainly corresponding to ‘catalytic activity’, ‘binding’ and ‘transporter activity’, while 15.85% of DEGs mapped to the ‘cellular component’ domain, predominantly including the terms ‘membrane’ and ‘membrane part’ (Fig. 8C). In addition, we also analyzed the DEGs that showed an opposite gene expression pattern during drought and recovery. The DEGs were significantly enriched in three KEGG pathways, including alpha-linolenic acid metabolism and galactose metabolism, indicating that genes involved in metabolism are notably affected by drought and recovery (Fig. 8D). GO analysis showed that 47.40% of DEGs mapped to the ‘biological process’ domain, with predominant terms including ‘metabolic process’, ‘single-organism process’, ‘cellular process’ and ‘response to stimulus’. Approximately 30% of these DEGs mapped to the domain ‘molecular function’, with terms mainly corresponding to ‘catalytic activity’, ‘binding’ and ‘transporter activity’, while 22.92% of the DEGs mapped to the ‘cellular component’ domain, with predominant terms including ‘membrane, ‘cell’ and ‘cell part’ (Fig. 8E).

### Modulation of transcription factor expression in *35 S::eGFP* and *AhGLK1-OX* hairy root during drought stress and post-drought recovery

**Fig. 9:**
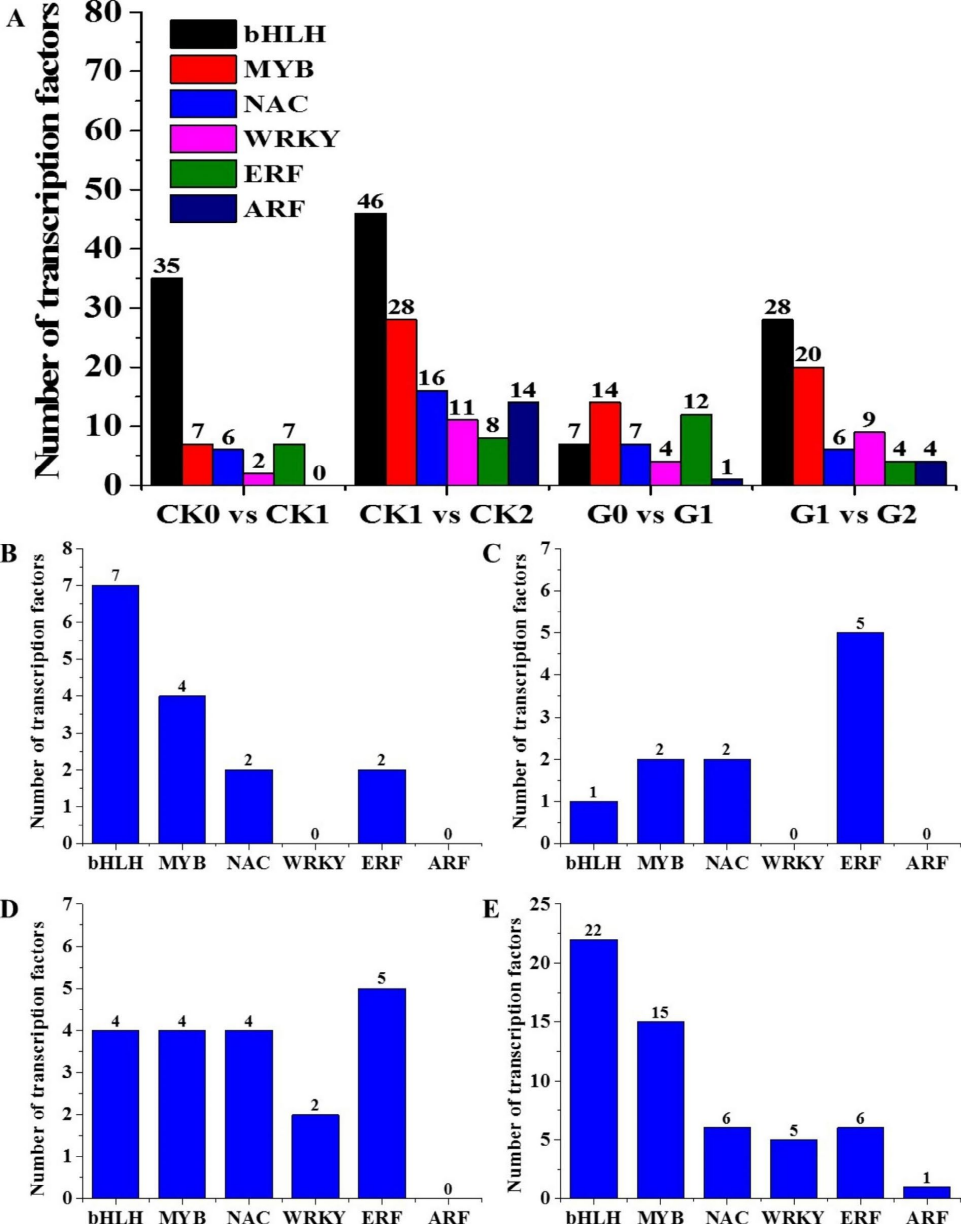
**Dynamics of the modulation of transcription factors during drought stress and post-drought recovery indicate differential regulation patterns in*35S::eGFP*and*AhGLK1-OX*hairy root.** (A) Differentially expressed TF gene families in the four pairwise groups. (B) Differentially expressed TF gene families in *35 S::eGFP* HR common to the response to drought and post-drought recovery. (C) Differentially expressed TF gene families in *AhGLK1-OX* HR common to the response to drought and post-drought recovery. (D) Differentially expressed TF gene families common to the drought-stress response in both *35 S::eGFP* and *AhGLK1-OX* HR. (E) Differentially expressed TF gene families common to the post-drought recovery process in both *35 S::eGFP* and *AhGLK1-OX* HR.

Transcription factors (TFs) play crucial roles in modulating physiological programs so that plants can adjust to changes in environmental conditions, including drought stress and post-drought recovery. To explore the complexity of regulatory mechanisms both in drought and recovery, differentially expressed TFs were examined in HR. A large number of genes belonging to the bHLH, MYB, NAC, WRKY, ERF and ARF families showed differential expression during drought stress and recovery compared to controls. For *35 S::eGFP* HR, 57 and 123 TF-encoding genes responded to drought stress and post-drought recovery, respectively. Among these, bHLH-family TFs were the most abundant, followed by MYB-family TFs. We identified 45 and 71 TF DEGs from the pairwise comparisons G0 vs. G1 and G1 vs. G2, respectively (Fig. 9A). The results imply that TFs, particularly from the bHLH and MYB families, play an important role in the response to drought stress and recovery both in *35 S::eGFP* and *AhGLK1-OX* HR.

Subsequently, we found 15 differentially expressed TFs in *35 S::eGFP* HR that were common to the response to drought and post-drought recovery, mostly from the bHLH and MYB families (Fig. 9B). For *AhGLK1-OX* HR, there were 10 differentially expressed TFs involved in both drought and the recovery process, but these were predominantly from the ERF family (Fig. 9C). In addition, there were 19 TFs involved in both types of HR during drought stress (Fig. 9D), of which ERF TFs were the most abundant. For post-drought recovery, 55 TFs were differentially expressed in both types of HR (Fig. 9E). Most of these were bHLH and MYB TFs, suggesting that these two TF families play a key role in the recovery process.

### Validation of gene expression by quantitative real-time PCR (qRT-PCR)

**Fig. 10:**
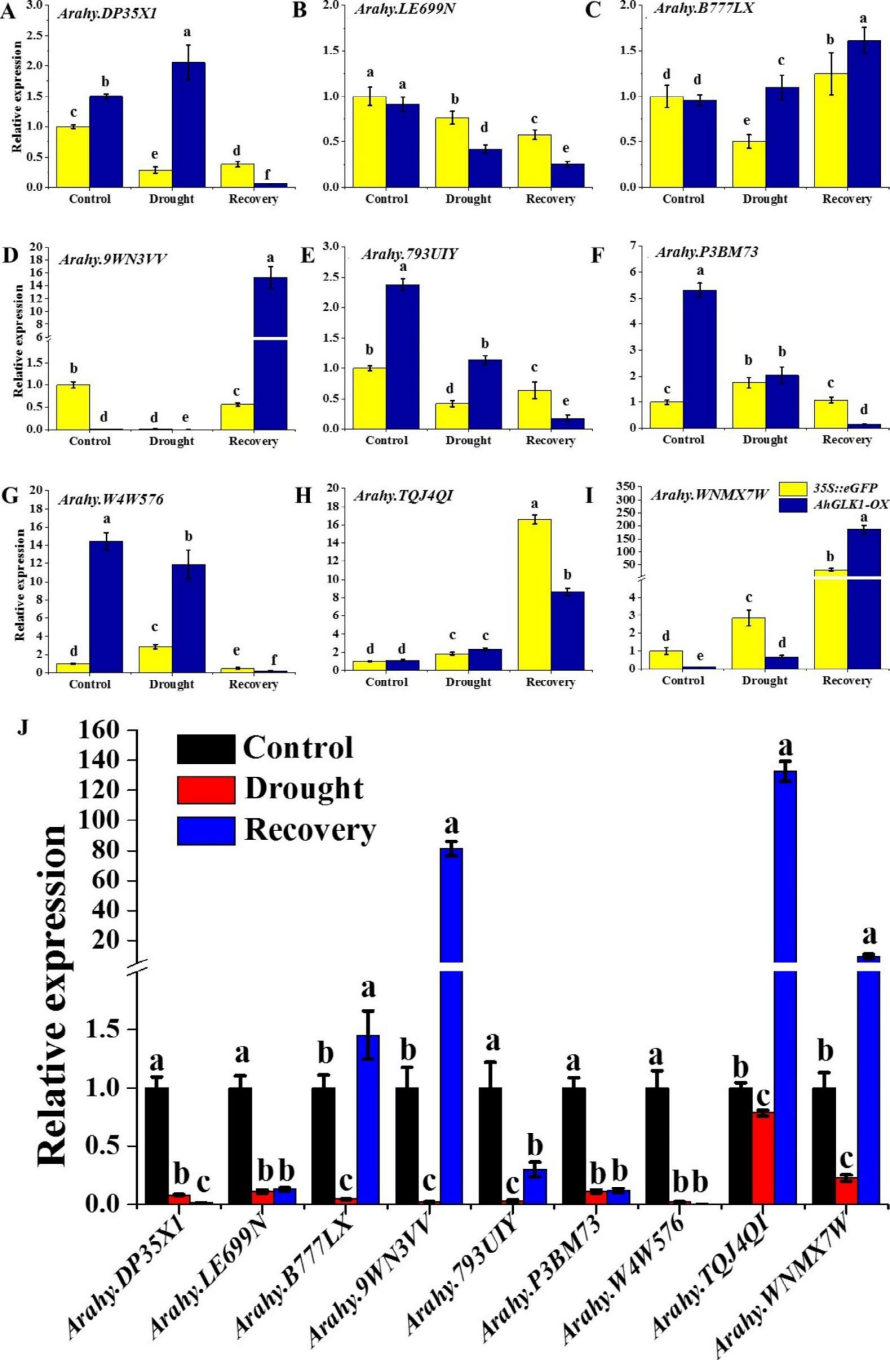
**Relative expression of DEGs analyzed by qRT-PCR in different hairy root and peanut leaf tissues**. (A ~ I) Gene expression level of *35 S::eGFP* and *AhGLK1-OX* HR under drought stress and post-drought recovery conditions. (J) Gene expression level in peanut leaf under drought stress and post-drought recovery conditions. The average of three biological replicates is shown. Error bars represent SE. Different letters (a, b, c and d) represent significantly different groups

To validate the results of RNA-seq and confirm how the expression of various DEGs changes in response to drought and recovery, we selected nine genes from some of the highly enriched KEGG pathways to perform a qRT-PCR analysis. The nine genes were as follows: *Arahy.DP35 × ****1***, encoding a MYB transcription factor; *Arahy.B777LX* and *Arahy.793UIY*, encoding fatty acyl-CoA reductase 3-like; *Arahy.P3BMC7* and *Arahy.LE699N* encoding cytochrome P450 superfamily protein; *Arahy.TQJ4QI*, encoding disease-resistance response protein; *Arahy.W4W576*, encoding O-methyltransferase family protein; *Arahy.WNMX7W*, encoding putative indole-3-acetic acid-amido synthetase; and *Arahy.9WN3VV*, encoding expansin-like B1. During drought, the expression level of three genes, *Arahy.LE699N*, *Arahy.9WN3VV* and *Arahy.793UIY*, was reduced, while expression of *Arahy.TQJ4QI* and *Arahy.WNMX7W* was increased both in *35 S::eGFP* and *AhGLK1-OX* HR. However, the expression level of *Arahy.793UIY* and *Arahy.B777LX* was significantly reduced in *35 S::eGFP*, but increased in *AhGLK1-OX* HR. In contrast, *Arahy.P3BMC7* and *Arahy.W4W576* expression was significantly increased in *35 S::eGFP*, but reduced in *AhGLK1-OX* HR. During post-drought recovery, *Arahy.B777LX*, *Arahy.9WN3VV*, *Arahy.TQJ4QI* and *Arahy.WNMX7W* expression was notably increased, while *Arahy.LE699N*, *Arahy.P3BMC7* and *Arahy.W4W576* expression was reduced in both types of HR. Interestingly, the expression level of *Arahy.793UIY* and *Arahy.DP35 × 1* was increased in *35 S::eGFP*, but reduced in *AhGLK1-OX* HR. The results show that the activity of these genes varied dynamically in response to drought and recovery (Fig. 10A-I). Subsequently, the expression of the above nine genes was assessed in peanut leaves. As shown in Fig. 10J, expression levels of all genes were significantly decreased in drought stress, indicating that they are inhibited by drought stress in peanut leaves. During recovery, however, the expression level of *Arahy.9WN3VV*, *Arahy.B777LX*, *Arahy.TQJ4QI* and *Arahy.WNMX7W* was significantly upregulated. In contrast, expression of the other five genes remained low, similar to the levels observed under drought conditions. Indeed, in some cases, such as *Arahy.DP35 × 1*, expression was even lower than that observed during drought. Thus, these peanut genes respond in a variety of ways depending on the environmental conditions, i.e. drought or post-drought recovery, and the tissue, i.e. HR or leaves. The qRT-PCR results show similar trends to those revealed by the RNA-seq data, giving further credence to our sequencing results.

## Discussion

In plants, the root is the first organ to sense drought stress in the soil. HR has been successfully induced from various explants, such as leaves, petiole and epicotyl, with the highest induction rates being from leaf explants. Therefore, we might ask what kind of differences exist in the anatomy of HR and PR. To address this, the morphology and transcriptomes of peanut HR and PR were compared in this study. It was previously reported that the root tips in HR were thicker and more robust than in the seedling roots of *Cassia obtusifolia*; the smaller epidermal cells and the cortex cells of HR were more closely and irregularly arranged [9]. As for peanut, HR also develops a root cap, meristem and elongation zone like in PR. However, unlike in PR, the cells in the HR root cap, apical meristem and cortex are inhomogeneous in size. They also show a difference in the adsorption of methylene blue, which stains plant tissue through a combination of physical adsorption and chemical affinity. After methylene blue staining, peanut PR appears purple-red, while HR becomes a deep purple-blue color. These results suggest there are crucial differences in the constitution of the cytoplasm or the pH between HR and PR in peanut. Nevertheless, HR originating from leaf explants develops a similar root structure to PR. These findings indicate that HR cultures are suitable for investigating gene functions involved in root development and stress responses.

A significant difference between the two root tissues emerged when we screened for differentially expressed genes. It was found that 516 DEGs were upregulated in HR compared to PR, and 88 of these were related to photosynthesis and chlorophyll synthesis. We also found that HR gradually turn green over time. In contrast, PR does not become green when cultivated on MS medium in the light. Thus, it seems that HR has the potential to synthesize chlorophyll, like leaves, suggesting that HR can be used to study genes that are mainly expressed in leaf. We also found that the plant hormone signal transduction pathway was enriched in downregulated DEGs, perhaps indicating that HR is less sensitive to the external environment than PR or that HR is better able to tolerate changes in the external environment. Our morphological and transcriptomics study reveals such differences between HR and PR for the first time.

Climate change is predicted to increase the severity and duration of drought events in many regions. Drought adversely influences plant growth and continues to threaten the world’s food security [31]. Plants acclimatize to drought by regulating their physiological and biochemical characteristics. In particular, post-drought recovery growth is important for enhancing the quality and yield of crops. In peanut, substantial crop losses occur worldwide each year due to a variety of abiotic stresses [32]. Peanut is predominantly grown in arid and semi-arid regions and its quality and yield are frequently impacted by drought stress; thus, post-drought recovery is critical for enhancing yield. However, no data are available on the genetic regulation of drought stress and post-drought recovery in peanut. In-depth research on this phenomenon will contribute to the development of molecular breeding strategies aimed at improving peanut drought tolerance and increasing its yield.

In this study, we revealed the various gene networks that are involved in the response to drought and post-drought recovery using HR. Our results show that, during drought, the flavonoid biosynthesis and circadian rhythm–plant pathways were the most significantly enriched in DEGs. Flavonoids are beneficial in that they improve the antioxidant capacity of plants and help alleviate damage caused by drought. Accordingly, our data suggest that HR protects itself from drought damage by synthesizing flavonoids. The plant circadian clock is a conserved regulatory mechanism that stimulates and maintains rhythmic elements of plant physiology [33]. The circadian clock helps to improve environmental adaptation and to ensure optimal plant growth and performance. Many key genes of the circadian clock are involved in the regulation of stress responses [34]. A recent study in rice found that the regulatory relationship between the expression of cell dehydration-responsive genes and circadian clock genes optimizes the water stress response at different times of the day, demonstrating that this mechanism plays an important role in increasing the survival rate and production efficiency of rice in an arid environment [35]. Hence, we speculate that HR also improves its ability to survive by altering circadian rhythms during drought stress. However, during post-drought recovery, 1784 DEGs were downregulated in both *35 S::eGFP* and *AhGLK1-OX* HR, while only 673 DEGs were upregulated in both types of HR, implying that a large number of DEGs show decreased expression during recovery. KEGG pathway analysis showed that metabolic pathways were the most significantly enriched in upregulated DEGs, with 108 in total. Thus, HR adapts to recovery growth by changing its metabolism, which is consistent with other research [36]. In contrast, downregulated DEGs were significantly enriched in starch and sucrose metabolism, phenylpropanoid biosynthesis, amino sugar and nucleotide sugar metabolism and plant hormone signal transduction, suggesting that the activity of these pathways was repressed during post-drought recovery.

Interestingly, when we looked for DEGs that were implicated in both the response to drought and post-drought recovery, we found 74 examples, which included three and six upregulated and downregulated DEGs, respectively, that were common to both types of HR. Of these, 65 DEGs were upregulated during drought, but downregulated during recovery, showing that these genes are regulated in a reciprocal fashion and thus may play contrasting roles during drought and recovery. Metabolic pathways and the biosynthesis of secondary metabolism pathway were enriched in these genes, showing that metabolic adjustment is the most important factor in the adaptation of HR to water deficit. We found that 20 DEGs that were uniquely expressed in *AhGLK1-OX* HR, but commonly expressed in both types of HR in recovery, were enriched in the photosynthesis-antenna proteins and photosynthesis, and porphyrin metabolism pathways (Fig. 6E). This suggests that photosynthesis and chlorophyll synthesis are important in *AhGLK1-OX* HR in response to changes in the external environment, consistent with a previous study [7].

TFs are essential in plant signal transduction pathways and many TF families, such as MYB, bZIP, bHLH, WRKY, NAC, ERF and DREB, are involved in the response to drought stress as key regulators [37]. In agreement with these studies, we found that members of the MYB, bHLH, NAC, WRKY, ERF and ARF families show differential expression in response to drought and post-drought recovery. Of these, bHLH TFs were the most abundant, implying that bHLH TFs play a critical role in the response to drought stress and recovery both in *35 S::eGFP* and *AhGLK1-OX* HR, consistent with previous reports showing that bHLH TFs are widely involved in the plant response to abiotic stresses, such as drought, cold and salinity [38].

## Conclusion

Our study explores the differences between HR and PR in terms of root structure and transcriptome. The results reveal that HR possesses at least some of the characteristics of leaf, indicating that HR is suitable for studying the function of genes mainly expressed in leaf. This should yield reliable experimental evidence for analysis of the differences between HR and PR. Analysis of how the HR transcriptome changes during drought and post-drought recovery provides insight into the regulatory networks operating in peanut during exposure to and recovery from water deficit. In addition, the analysis reveals the gene networks specifically regulated by *AhGLK1* in response to drought and recovery, elucidating the molecular mechanisms by which *AhGLK1* improves drought resistance and post-drought recovery ability in peanut. However, the candidate genes that play the most important roles during drought and recovery require further study. In conclusion, this study provides a theoretical basis and a new perspective for in-depth exploration of drought resistance genes in peanut, and will be invaluable for molecular breeding of more resistant varieties.

## Materials and methods

### Plant materials and growth, and hairy root transformation

The South China peanut cultivar ‘Yueyou7’ was used in this study. The seeds were kindly provided by Dr. Xuanqiang Liang from the Crop Research Institute, Guangdong Academy of Agricultural Sciences, Guangzhou. The study was carried out in compliance with institutional guidelines. The *p35S*::*eGFP* and *p35S::AhGLK1-eGFP* constructs were transformed into *Agrobacterium rhizogenes* strain K599 and used to induce the production of HR in peanut leaves according to a method established previously in our laboratory. HR induced by the *p35S*::*eGFP* plasmid was used as the blank control, and is denoted *35 S::eGFP* here. HR overexpressing *AhGLK1* was induced by the *p35S::AhGLK1-eGFP* plasmid, and is denoted *AhGLK1-OX*. Peanut plants were grown under normal conditions as described previously [39]. Seedlings were grown in a greenhouse at 25–28 °C. Leaves from 10-12-day-old plants were collected and prepared for explant as previously [40]. Leaf explants were cut with a blade to generate wounds and then immersed in *A. rhizogenes* (K599) suspension (OD ≈ 0.5) for 10 min. The infected leaf explants were dried on sterile filter paper and transferred to solid MS medium for 48 h in the dark. After co-cultivation, the explants were sterilized with 250 mg.L^− 1^ cefotaxime (Dingguo, Guangzhou, China) for 5 min and washed with sterile water three times. All explants were then cultured on solid MS medium containing 250 mg.L^− 1^ cefotaxime. HR was induced at the wound sites over a period of about four weeks.

### Root sections and staining

About 0.5 ~ 0.8 centimeter root tips from HR about 30-day-old or PR about 14-day-old were fixed in F.A.A fixative (5 mL 38% formalin, 5 mL acetic acid, 90 mL 70% alcohol) for about 30 min through vacuum infiltration. The specimens were dehydrated in successive ethanol-water mixtures for 15 min with increasing ethanol concentrations (30, 50, 70, 80, and 90%), followed by two 30 min dehydrations in 100% ethanol. Then, the root tips were treated with a 1:1 mixture of anhydrous ethanol and xylene for 30 min, and xylene for 1 h. After that, the root tips were embedded in paraffin blocks and cut into consecutive Sect. (5 μm thick slices) using a microtome, then placed onto glass slides. The sections were dewaxed with different concentrations of xylene, and after immersion in a gradient of alcohol (high concentration to low concentration, 3 min for each time), finally washed with distilled water, 5 min per time. Sections of root tip were stained with methylene blue for 3 min, then washed with distilled water for three times. The sections were observed and photographed with an upright metallurgical microscope (DM6, Leica, Germany).

### Phenotype, relative water content and chlorophyll content of hairy root

HR grown for about 30 days was harvested to determine the phenotype; chloroplasts were observed by confocal microscope. Drought samples of *35 S::eGFP* and *AhGLK1-OX* HR were generated by dehydration for 2 h in an incubator. For post-drought recovery samples, HR that had been dehydrated for 2 h was re-watered for 2 h. Fresh HR (100 mg) was harvested from control, drought and recovery tissue. The relative water content and chlorophyll (*a* and *b*) concentration of the samples were determined as described previously [30].

### Transcriptome analysis and validation

For transcriptome preparation, PR from 14 day-old peanut seedlings, and 30 day-old *35 S::eGFP* and *AhGLK1-OX* HR, were harvested to extract total RNA. HR was divided into three groups comprising a control that was not dehydrated, a drought group that was dehydrated for 2 h in an incubator and a recovery group that was dehydrated for 2 h then re-watered for 2 h. Total RNAs were extracted from these HR sample using TRIzol. The samples were denoted CK0 (control conditions, *35 S::eGFP* HR), CK1 (drought, *35 S::eGFP* HR), CK2 (recovery, *35 S::eGFP* HR), G0 (control, *AhGLK1-OX* HR), G1 (drought, *AhGLK1-OX* HR) and G2 (recovery, *AhGLK1-OX* HR). RNA concentration was measured initially on a NanoDrop 2000 and more precise measurements were made on an Agilent 2100 Bioanalyzer. Next, mRNA was enriched using an oligo(dT) resin. Second-strand cDNA was synthesized by DNA polymerase I, RNase H, dNTP and buffer, and purified with a QIAquik PCR Purification Kit (Qiagen), then end-repaired followed by addition of poly(A). Purified cDNA was ligated to Illumina sequencing adapters. Sequencing was carried out using an Illumina HiSeq 4000 system by Gene Denovo Biotechnology Co (Guangzhou, China). All raw data have been submitted to the NCBI Sequence Read Archive (SRA) (https://www.ncbi.nlm.nih.gov/bioproject/PRJNA503795 and https://www.ncbi.nlm.nih.gov/bioproject/PRJNA580047).

For bioinformatics analysis, raw reads were first filtered to remove rRNA and low quality sequences. High-quality clean reads were mapped to a reference transcriptome and gene abundance was calculated and normalized to RPKM (reads per kb per million reads). Differentially expressed genes (DEGs) were identified as those with gene abundance ≥ 100, fold change ≥ 2 compared to the reference and a false discovery rate (FDR) < 0.05. DEGs were subjected to gene ontology (GO) enrichment analysis (www.geneontology.org/) and were mapped to Kyoto Encyclopedia of Genes and Genomes (KEGG) pathways (www.genome.jp/kegg/) [41]. The heatmap was generated using the PageMan tool with average statistics and Benjamini-Hochberg multiple testing correction for all the significant differentially regulated genes from the various HR samples with fold change (± 1.5) and p-value ≤ 0.05 [42].

Quantitative RT-PCR (qPCR) was conducted to validate DEGs. Total RNA was prepared as above and reversed-transcribed to cDNA using the PrimeScript™ RT Reagent Kit (Takara) according to the manufacturer’s instructions. qPCR analyses were performed as previously described [30]. The cDNA and primers were diluted for PCR, and *AhActin* was used as the reference gene.

## Electronic supplementary material

Below is the link to the electronic supplementary material.

**Additional file 1: Supplemental Dataset 1.** List of DEGs up-regulated in HR compared to PR. **Supplemental Dataset 2.** List of DEGs down-regulated in HR compared to PR. **Supplemental Dataset 3.** List of DEGs up- and down-regulated in CK1 compared to CK0. **Supplemental Dataset 4.** List of DEGs up- and down-regulated in G1 compared to G0. **Supplemental Dataset 5.** List of common up-regulated DEGs in CK0 VS CK1 and G0 VS G1. **Supplemental Dataset 6.** List of common down-regulated DEGs in CK0 VS CK1 and G0 VS G1. **Supplemental Dataset 7.** List of DEGs up- and down-regulated in CK2 compared to CK1.** Supplemental Dataset 8.** List of DEGs up- and down-regulated in G2 compared to G1. **Supplemental Dataset 9.** List of common up-regulated DEGs in CK1 VS CK2 and G1 VS G2. **Supplemental Dataset 10.** List of common down-regulated DEGs in CK1 VS CK2 and G1 VS G2. **Supplemental Dataset 11. **List of 74 DEGs both in drought stress and post-drought recovery process. **Supplemental Dataset 12**. List of 20 DEGs uniquely expressed in AhGLK1-OX hairy root but common expressed in drought and recovery. **Supplemental Dataset 13.** List of DEGs of 35S::eGFP hair roots in response to drought and post-drought recovery. **Supplemental Dataset 14. **List of DEGs of AhGLK1-OX hair roots in response to drought and post-drought recovery.

## Data Availability

All data generated or analyzed for this study are included in this article and its supplementary files. The raw sequence reads were deposited into NCBI SRA database under accession no. PRJNA503795 and 580,047 (https://www.ncbi.nlm.nih.gov/bioproject/PRJNA503795 and https://www.ncbi.nlm.nih.gov/bioproject/PRJNA580047).

## References

[1] Luo H, Guo J, Ren X, Chen W, Huang L, Zhou X, Chen Y, Liu N, Xiong F, Lei Y (2018). Chromosomes A07 and A05 associated with stable and major QTLs for pod weight and size in cultivated peanut (Arachis hypogaea L). Theor Appl Genet.

[2] Guimaraes PM, Brasileiro AC, Morgante CV, Martins AC, Pappas G, Silva OB, Togawa R, Leal-Bertioli SC, Araujo AC, Moretzsohn MC (2012). Global transcriptome analysis of two wild relatives of peanut under drought and fungi infection. BMC Genomics.

[3] Kiranmai K, Lokanadha Rao G, Pandurangaiah M, Nareshkumar A, Amaranatha Reddy V, Lokesh U, Venkatesh B, Anthony Johnson AM, Sudhakar C (2018). A novel WRKY transcription factor, MuWRKY3 (Macrotyloma uniflorum Lam. Verdc.) Enhances Drought stress tolerance in transgenic groundnut (Arachis hypogaea L.) plants. Front Plant Sci.

[4] Sinharoy S, Saha S, Chaudhury SR, Dasgupta M (2009). Transformed hairy roots of Arachis hypogea: a tool for studying root nodule symbiosis in a non-infection thread legume of the Aeschynomeneae tribe. Mol Plant Microbe Interact.

[5] Liu S, Su L, Liu S, Zeng X, Zheng D, Hong L, Li L (2016). Agrobacterium rhizogenes-mediated transformation of Arachis hypogaea: an efficient tool for functional study of genes. Biotechnol Biotechnol Equip.

[6] Su L, Liu S, Liu X, Zhang B, Li M, Zeng L, Li L (2021). Transcriptome profiling reveals histone deacetylase 1 gene overexpression improves flavonoid, isoflavonoid, and phenylpropanoid metabolism in Arachis hypogaea hairy roots. PeerJ.

[7] Liu X, Li L, Zhang B, Zeng L, Li L (2020). AhHDA1-mediated AhGLK1 promoted chlorophyll synthesis and photosynthesis regulates recovery growth of peanut leaves after water stress. Plant Sci.

[8] Huet Y, Ekouna JP, Caron A, Mezreb K, Boitel-Conti M, Guerineau F (2014). Production and secretion of a heterologous protein by turnip hairy roots with superiority over tobacco hairy roots. Biotechnol Lett.

[9] Tan RH, Zhang JJ, Zhao SJ (2014). [Optimization of induction and culture conditions for hairy roots of Salvia miltiorrhiza]. Zhongguo Zhong Yao Za Zhi.

[10] Shirazi Z, Aalami A, Tohidfar M, Sohani MM (2018). Metabolic Engineering of Glycyrrhizin Pathway by Over-Expression of Beta-amyrin 11-Oxidase in transgenic roots of Glycyrrhiza glabra. Mol Biotechnol.

[11] Condori J, Sivakumar G, Hubstenberger J, Dolan MC, Sobolev VS, Medina-Bolivar F (2010). Induced biosynthesis of resveratrol and the prenylated stilbenoids arachidin-1 and arachidin-3 in hairy root cultures of peanut: Effects of culture medium and growth stage. Plant Physiol Biochem.

[12] Akhgari A, Yrjonen T, Laakso I, Vuorela H, Oksman-Caldentey KM, Rischer H (2015). Establishment of transgenic Rhazya stricta hairy roots to modulate terpenoid indole alkaloid production. Plant Cell Rep.

[13] Yang T, Fang L, Nopo-Olazabal C, Condori J, Nopo-Olazabal L, Balmaceda C, Medina-Bolivar F (2015). Enhanced production of Resveratrol, Piceatannol, Arachidin-1, and Arachidin-3 in Hairy Root cultures of peanut co-treated with Methyl Jasmonate and Cyclodextrin. J Agric Food Chem.

[14] Nedelkoska TV, Doran PM (2001). Hyperaccumulation of nickel by hairy roots of alyssum species: comparison with whole regenerated plants. Biotechnol Prog.

[15] Guimaraes LA, Pereira BM, Araujo ACG, Guimaraes PM, Brasileiro ACM (2017). Ex vitro hairy root induction in detached peanut leaves for plant-nematode interaction studies. Plant Methods.

[16] Chu Y, Guimaraes LA, Wu CL, Timper P, Holbrook CC, Ozias-Akins P (2014). A technique to study Meloidogyne arenaria Resistance in Agrobacterium rhizogenes-transformed peanut. Plant Dis.

[17] Chen D, Wang S, Cao B, Cao D, Leng G, Li H, Yin L, Shan L, Deng X (2015). Genotypic variation in growth and physiological response to drought stress and re-watering reveals the critical role of recovery in drought adaptation in maize seedlings. Front Plant Sci.

[18] Dong S, Jiang Y, Dong Y, Wang L, Wang W, Ma Z, Yan C, Ma C, Liu L (2019). A study on soybean responses to drought stress and rehydration. Saudi J Biol Sci.

[19] Lawas LMF, Erban A, Kopka J, Jagadish SVK, Zuther E, Hincha DK (2019). Metabolic responses of rice source and sink organs during recovery from combined drought and heat stress in the field. Gigascience.

[20] Cao L, Lu X, Wang G, Zhang P, Fu J, Wang Z, Wei L, Wang T (2021). Transcriptional regulatory networks in response to drought stress and rewatering in maize (Zea mays L). Mol Genet Genomics.

[21] Wang Y, Yang Z, Shi L, Yang R, Guo H, Zhang S, Geng G (2022). Transcriptome analysis of Auricularia fibrillifera fruit-body responses to drought stress and rehydration. BMC Genomics.

[22] Waters MT, Wang P, Korkaric M, Capper RG, Saunders NJ, Langdale JA (2009). GLK transcription factors coordinate expression of the photosynthetic apparatus in Arabidopsis. Plant Cell.

[23] Cackett L, Luginbuehl LH, Schreier TB, Lopez-Juez E, Hibberd JM (2022). Chloroplast development in green plant tissues: the interplay between light, hormone, and transcriptional regulation. New Phytol.

[24] Ni F, Wu L, Wang Q, Hong J, Qi Y, Zhou X (2017). Turnip yellow mosaic virus P69 interacts with and suppresses GLK transcription factors to cause pale-green symptoms in Arabidopsis. Mol Plant.

[25] Townsend PD, Dixon CH, Slootweg EJ, Sukarta OCA, Yang AWH, Hughes TR, Sharples GJ, Palsson LO, Takken FLW, Goverse A (2018). The intracellular immune receptor Rx1 regulates the DNA-binding activity of a Golden2-like transcription factor. J Biol Chem.

[26] Nagatoshi Y, Mitsuda N, Hayashi M, Inoue S-i, Okuma E, Kubo A, Murata Y, Seo M, Saji H, Kinoshita T (2016). GOLDEN 2-LIKE transcription factors for chloroplast development affect ozone tolerance through the regulation of stomatal movement. Proc Natl Acad Sci USA.

[27] Ahmad R, Liu Y, Wang TJ, Meng Q, Yin H, Wang X, Wu Y, Nan N, Liu B, Xu ZY (2019). GOLDEN2-LIKE transcription factors regulate WRKY40 expression in response to abscisic acid. Plant Physiol.

[28] Nguyen CV, Vrebalov JT, Gapper NE, Zheng Y, Zhong S, Fei Z, Giovannoni JJ (2014). Tomato GOLDEN2-LIKE transcription factors reveal molecular gradients that function during fruit development and ripening. Plant Cell.

[29] Li X, Wang P, Li J, Wei S, Yan Y, Yang J, Zhao M, Langdale JA, Zhou W (2020). Maize GOLDEN2-LIKE genes enhance biomass and grain yields in rice by improving photosynthesis and reducing photoinhibition. Commun Biol.

[30] Liu X, Li L, Li M, Su L, Lian S, Zhang B, Li X, Ge K, Li L (2018). AhGLK1 affects chlorophyll biosynthesis and photosynthesis in peanut leaves during recovery from drought. Sci Rep.

[31] Liu C, Wang Y, Pan K, Wang Q, Liang J, Jin Y, Tariq A (2017). The synergistic responses of different Photoprotective Pathways in dwarf bamboo (Fargesia rufa) to Drought and subsequent rewatering. Front Plant Sci.

[32] Mou Y, Sun Q, Yuan C, Zhao X, Wang J, Yan C, Li C, Shan S (2022). Identification of the LOX Gene Family in Peanut and functional characterization of AhLOX29 in Drought Tolerance. Front Plant Sci.

[33] Chow BY, Kay SA (2013). Global approaches for telling time: omics and the Arabidopsis circadian clock. Semin Cell Dev Biol.

[34] Ding YE, Zou YN, Wu QS, Kuca K (2022). Mycorrhizal fungi regulate daily rhythm of circadian clock in trifoliate orange under drought stress. Tree Physiol.

[35] Usman B, Nawaz G, Zhao N, Liao S, Liu Y, Li R. Precise Editing of the OsPYL9 Gene by RNA-Guided Cas9 Nuclease Confers Enhanced Drought Tolerance and Grain Yield in Rice (Oryza sativa L.) by Regulating Circadian Rhythm and Abiotic Stress Responsive Proteins. Int J Mol Sci 2020, 21(21). 10.3390/ijms21217854 PMC7660227 33113937

[36] Shi R, Jiao W, Yun L, Zhang Z, Zhang X, Wang Q, Li Y, Mi F (2021). Utilization of Transcriptome, small RNA, and Degradome sequencing to provide Insights into Drought stress and Rewatering Treatment in Medicago ruthenica. Front Plant Sci.

[37] Singh D, Laxmi A (2015). Transcriptional regulation of drought response: a tortuous network of transcriptional factors. Front Plant Sci.

[38] Qian Y, Zhang T, Yu Y, Gou L, Yang J, Xu J, Pi E (2021). Regulatory Mechanisms of bHLH transcription factors in Plant adaptive responses to various Abiotic stresses. Front Plant Sci.

[39] Li X, Lu J, Liu S, Liu X, Lin Y, Li L (2014). Identification of rapidly induced genes in the response of peanut (Arachis hypogaea) to water deficit and abscisic acid. BMC Biotechnol.

[40] Liu S, Li M, Su L, Ge K, Li L, Li X, Liu X, Li L (2016). Negative feedback regulation of ABA biosynthesis in peanut (Arachis hypogaea): a transcription factor complex inhibits AhNCED1 expression during water stress. Sci Rep.

[41] Kanehisa M, Goto S (2000). KEGG: kyoto encyclopedia of genes and genomes. Nucleic Acids Res.

[42] Usadel B, Nagel A, Steinhauser D, Gibon Y, Blasing OE, Redestig H, Sreenivasulu N, Krall L, Hannah MA, Poree F (2006). PageMan: an interactive ontology tool to generate, display, and annotate overview graphs for profiling experiments. BMC Bioinformatics.

